# Estimating Memory Deterioration Rates Following Neurodegeneration and Traumatic Brain Injuries in a Hopfield Network Model

**DOI:** 10.3389/fnins.2017.00623

**Published:** 2017-11-09

**Authors:** Melanie Weber, Pedro D. Maia, J. Nathan Kutz

**Affiliations:** Department of Applied Mathematics, University of Washington, Seattle, WA, United States

**Keywords:** neurodegenerative diseases, traumatic brain injuries, Hopfield neuronal network, associative memory encoding, memory impairments, axonal swellings

## Abstract

Neurodegenerative diseases and traumatic brain injuries (TBI) are among the main causes of cognitive dysfunction in humans. At a neuronal network level, they both extensively exhibit focal axonal swellings (FAS), which in turn, compromise the information encoded in spike trains and lead to potentially severe functional deficits. There are currently no satisfactory quantitative predictors of decline in memory-encoding neuronal networks based on the impact and statistics of FAS. Some of the challenges of this translational approach include our inability to access small scale injuries with non-invasive methods, the overall complexity of neuronal pathologies, and our limited knowledge of how networks process biological signals. The purpose of this computational study is three-fold: (i) to extend Hopfield's model for associative memory to account for the effects of FAS, (ii) to calibrate FAS parameters from biophysical observations of their statistical distribution and size, and (iii) to systematically evaluate deterioration rates for different memory-recall tasks as a function of FAS injury. We calculate deterioration rates for a face-recognition task to account for highly correlated memories and also for a discrimination task of random, uncorrelated memories with a size at the capacity limit of the Hopfield network. While it is expected that the performance of any injured network should decrease with injury, our results link, for the first time, the memory recall ability to observed FAS statistics. This allows for plausible estimates of cognitive decline for different stages of brain disorders within neuronal networks, bridging experimental observations following neurodegeneration and TBI with compromised memory recall. The work lends new insights to help close the gap between theory and experiment on how biological signals are processed in damaged, high-dimensional functional networks, and towards positing new diagnostic tools to measure cognitive deficits.

## 1. Introduction

Neurodegenerative diseases and traumatic brain injuries (TBI) are responsible for an overwhelming variety of functional deficits, both cognitive and behavioral, in animals and in humans. Memory impairment, which is the focus of this work, is a particularly pernicious consequence for those affected. The pathophysiology induced by these brain disorders is usually complex, with key effects of the injuries occurring at small spatial scales that are currently inaccessible by non-invasive diagnostic techniques. Indeed, a hallmark pathology is the abundance of diffuse, Focal Axonal Swellings (FAS) which compromise spike train encodings in neuronal networks. Thus, there is a broad need to understand how neuronal pathologies which develop at a cellular level compromise the functionality of a network of neurons responsible for cognitive function. In this article, we extend a well-established computational model of associative memory, i.e., the Hopfield network model (Hopfield, 1982, 1984), to incorporate FAS pathologies implicated in many brain disorders, providing novel metrics for quantifying memory impairments and functional deficits. While random neuron and synapse loss have been studied previously in memory models, this work is the first to integrate them with a recently developed theory of impaired neuronal responses due to FAS and their effects to spike train coding (Maia and Kutz, 2014a,b, 2017; Maia et al., 2015). While it is obvious that damaging any network will lead to compromised functionality, our quantification of memory decline progression could lead to new metrics for understanding memory impairments in a variety of leading neurodegenerative diseases.

Our computational models have several limitations and are of course only a simplified proxy for the original neural circuity involved in memory tasks. Still, they can provide an interesting tradeoff as we address questions that would be otherwise impossible in a clinical or experimental setting. We define unambiguously and in a precise mathematical way quantities such as memory overlap, successful memory recognition, memory confusion, significance of the memory classification, and noise-handling ability. These variables are all incorporated in our recognition score, a new metric that offers a more complete description of the network' s performance. We can then systematically increment the injury level, the noise level, randomize targeted neurons, change FAS parameter distributions, and address either highly correlated memory sets (faces) or random and uncorrelated ones. Consequently, we are able to quantify the decline in memory performance as a collective function of these variables. Moreover, we run enough simulations to control for each variable, achieve statistically significant results, and estimate the variability between different realizations. We show that as the injury progress, the first affected network functionality is its noise-handling ability, and that explicit memory-confusion occurs only later on. In this regard, the FAS parameters and distributions are crucial to determine *when* and *how fast* confusion in associative-memory should be expected.

The seminal work of John Hopfield is still largely used to model memory association (Hopfield, 1982, 1984; Hopfield and Tank, 1985). There, meaningful stimuli are encoded, stored, and later recalled in response to some cue. And although this process improves the interpretation of subsequent stimuli that share common features with previously stored concepts, what ultimately governs the memory recall abilities are the coordinated exchanges of electrical signals between neurons in network structures. Several pathological effects, most notably FAS, can jeopardize this critical electrical activity in the brain, making memory performance particularly sensitive to common disorders. Given its ubiquity across many neurodegenerative diseases and traumatic brain injury, understanding the role of FAS in altering spike train encodings is of paramount importance, particularly on a network level where cognitive functionality occurs. The purpose of this work is to help close this gap and study the effects of FAS to network models that exhibit plausible memory retrieval capabilities.

## 2. Results

Some of our contributions are theoretical and methodological as we combine, for the first, time two models: (a) Hopfield's network model for associative memory (Hopfield, 1982, 1984) and (b) the theory of impaired neuronal responses due to FAS (Maia and Kutz, 2014a,b, 2017; Maia et al., 2015). Full details are provided in the Materials and Methods section and in the supplemental materials. It is important to mention that the previous FAS results by Maia and Kutz concern individual swellings only. Given the geometrical parameters of a particular swelling, they were able to estimate the corresponding type of impaired neural response (transmission, filtering, reflection, or blockage). In other hand, Hopfield's memory model is a network model. To study injuries at its level, we must provide plausible distributions of swelling effects to the injured population. Thus, the FAS parameters (pie-charts) calibrated from the experiments of Wang et al. and of Dikranian et al. (detailed ahead) are also important methodological contributions of this work. In what follows, we will illustrate these ideas for two different memory-retrieval tasks: (i) a face recognition task for highly correlated memories, and (ii) a memory retrieval task for random, uncorrelated memories. For each task, we tried to justify our simulation parameters as much biologically and statistically as possible, but since there were still some arbitrary selections, we made all our codes available to allow further parameter explorations.

### 2.1. Face recognition decline on impaired associative-memory networks

**Figure 1:**
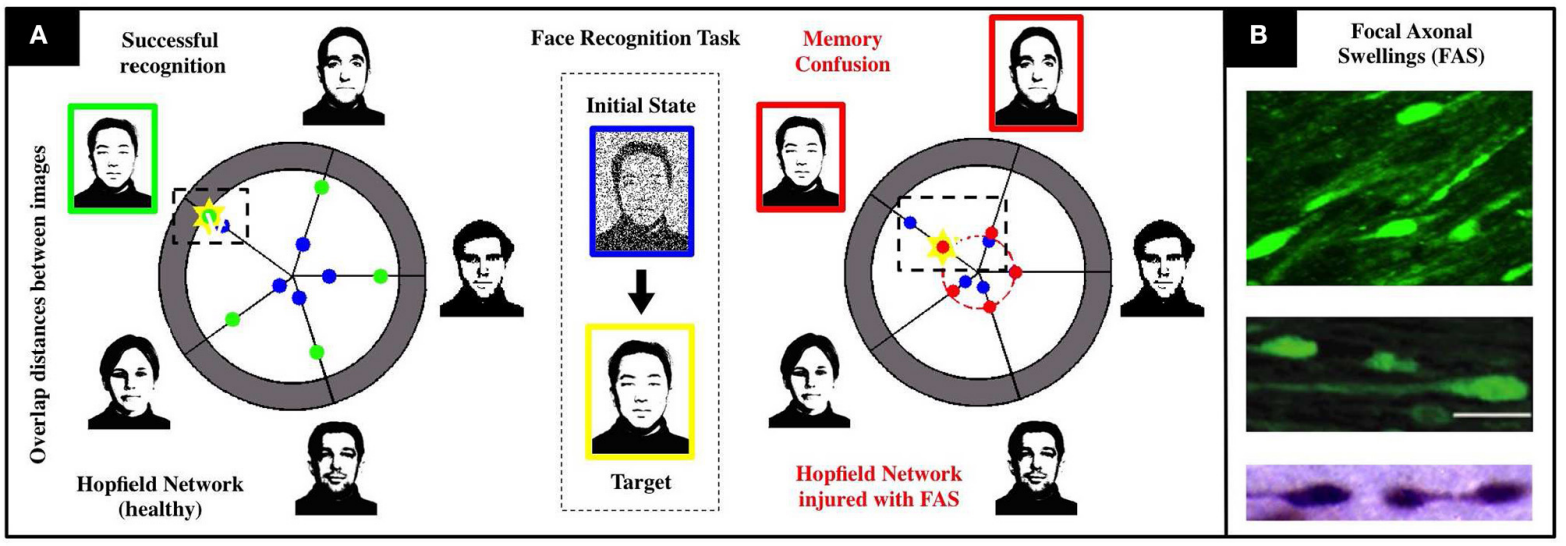
Schematics of our associative memory task (face-recognition) for a neuronal network under the influence of neurodegenerative diseases and traumatic brain injury. **(A)** For a noisy input with 80% overlap (i.e., 20% initial noise shown in center), the healthy system (left) achieves an overlap of 90% with the correct face (marked yellow). The chart presents the initial (blue) and final (green) Hamming distances between the current network state and the patterns corresponding to the respective facial images. When the recognition task is performed with an injured system (right), we observe a severe decrease in accuracy (overlap significantly smaller than in the healthy network) and confusion between two facial images. Both are characteristic injurious effects of memory impairments arising from FAS and are quantified with a recognition score in our study. **(B)** Images of FAS—highlighting their morphometric features—adapted from experimental works used to calibrate our model (Dikranian et al., 2008; Wang et al., 2011).

We calibrate a Hopfield-like neuronal network (Hopfield, 1982, 1984) to perform face recognition tasks. See Figure 1 for a detailed schematics. Our sample memory space consists of five human facial images (1, 044 × 1, 341 pixels), modified from the *MIT faces database* (Weyrauch et al., 2004)^1^. In our setting, neurons dynamically alternate between multiple activity states to account for multiple shades of grey in the images. This higher-dimensional feature space allows for the encoding and storing of more complex images in the network. The neuronal connections (re-weighted during an initial training phase) encode the desired memories as fixed points of the system. We add Brownian noise fluctuations to the dynamics as a proxy for natural stochastic fluctuations. A single realization of the face recognition task consists in presenting a different, noisy version of a memory and assess if the system converges to the corresponding fixed point. Our healthy network shown in Figure 1 converges to the right fixed point approximately 90% of the time, does not converge for the remaining 10%, and never converges to a wrong fixed point. Note that there are two types of errors (non-convergence and wrong-convergence). Our recognition score *R* (see Supplementary Materials) is a metric that encapsulates both types of errors and is better suited to account for deficits in network performance as FAS injuries are introduced in the simulations.

**Figure 2:**
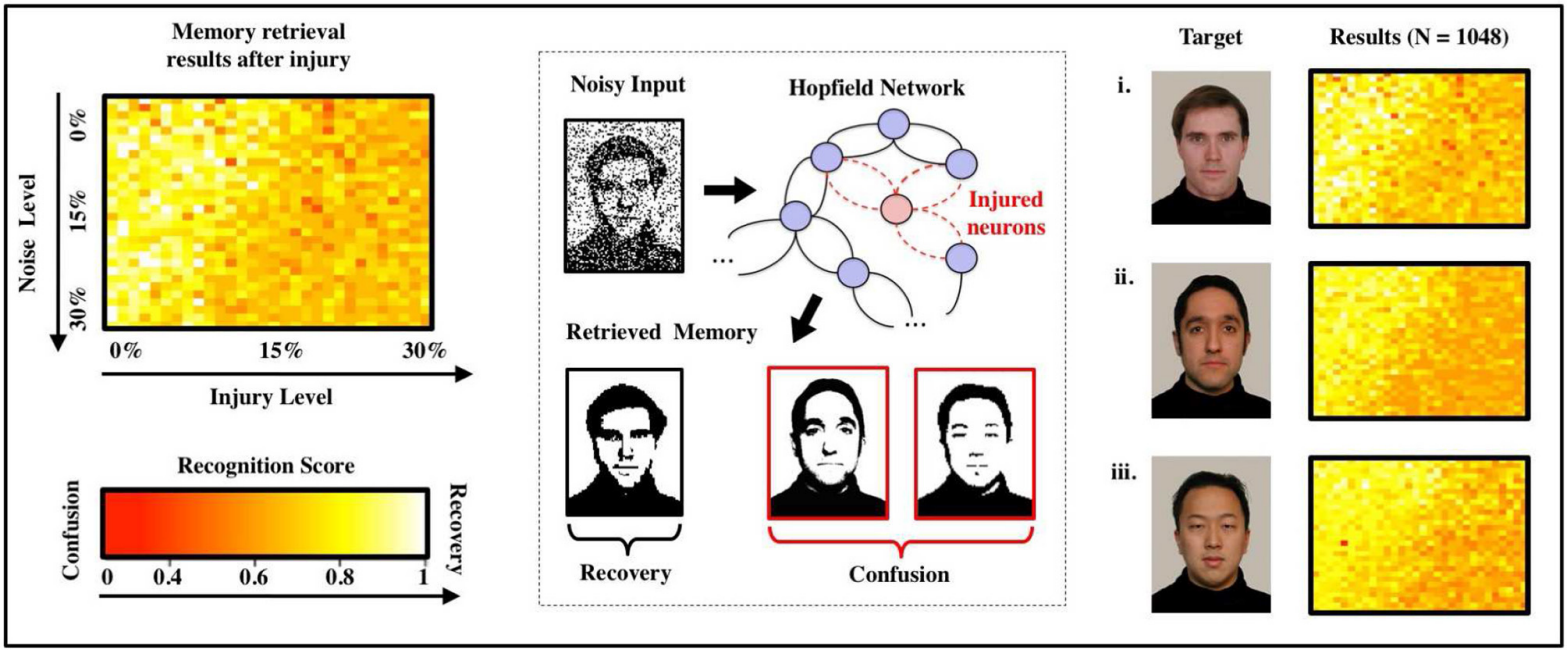
Effects of FAS on associative memory for a face recognition task. We measure recognition ability and accuracy for a sample set of three facial images (Weyrauch et al., 2004) over a given range of parameters (injury: 0–30%, initial noise: 0–30%). As the degree of injury increases, the noise handling ability of the system drops severely: The coloring of the heat maps change from yellow (significant recognition) in the upper left (small injury and initial noise) to dark orange and red (confusion) in the lower right corner (high injury and initial noise). We observe confusion and a decline in recall accuracy for every image in the set of samples. Regression over all *N* = 1,080 normalized data points indicates an exponentially declining relation between recognition scores and injury level as shown in the diagram.

Figure 2 shows recognition scores (heatmaps) for the most similar triplet of faces as we vary the noise level (from 0 to 30%) and the injury level *p* (from 0 to 30%). Note that recognition is always strong in the upper-left corner (low noise and low injury) and always weak in the lower-right corner (high noise and high injury). A careful examination of the heatmaps allow us to follow (for each face) how the decline in noise-handling ability evolves into memory confusion.

**Figure 3:**
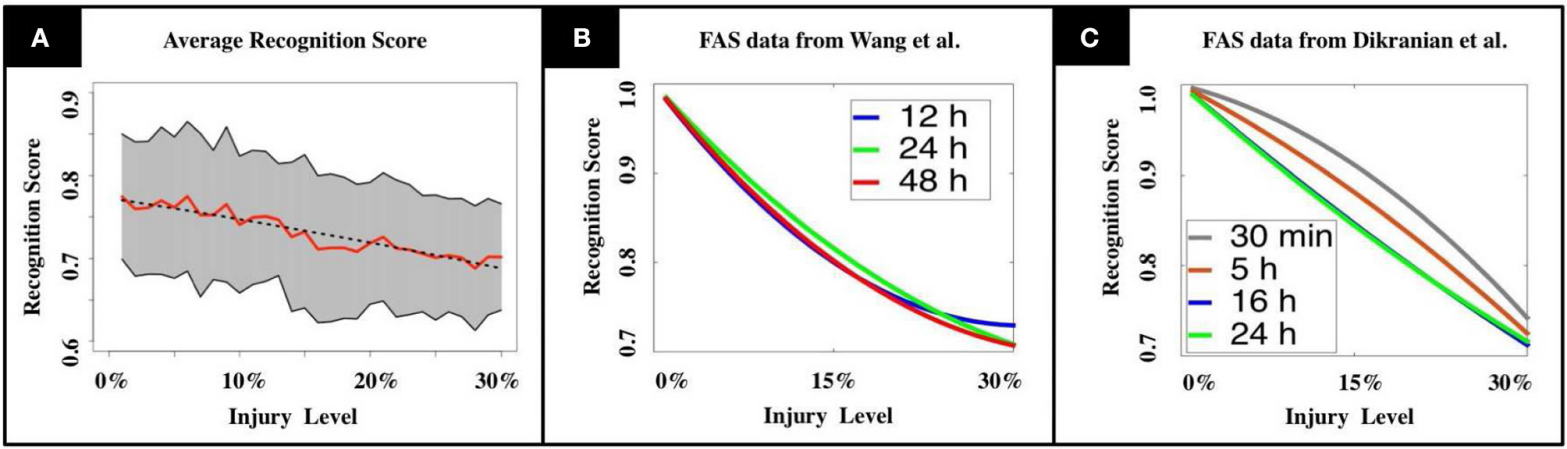
**(A)** Mean recognition scores are displayed in red, standard deviations are shaded in gray. The dashed line indicates the recognition function obtained by linear regression. **(B,C)** We tested our model using experimental results from TBI studies by Wang et al. (2011) and Dikranian et al. (2008). Our results show a modest, but unstable increase in memory performance for the adult brain during the first 24 h after the injury (**B**, Wang et al., 2011), that drops again to the initial level during the following day. In the infant's mouse brain, we observe a steadily decreasing performance in the first 24 h (**C**, Dikranian et al., 2008). The observations match the long recovering times that are commonly observed in patients suffering from TBI.

A key innovation of our model is that a swollen neuron may operate in one of the following regimes: total transmission, filtering, reflection, or blockage. The injury level *p* denotes the percentage of randomly injured neurons in the network, but their regimes are determined by a FAS distribution (pie-charts). The FAS pie-charts change depending on how long it took for the mice to be sacrificed. Figure 3A uses FAS pie-charts inferred experimentally (Wang et al., 2011) for mice sacrificed 12 h after TBI. Figure 3B uses pie-charts from mice sacrificed 24 and 48 h after TBI. Finally, Figure 3C uses pie-charts from another source (Dikranian et al., 2008), for mice sacrificed 30 min, 5, 16, and 24 h after TBI. This allowed us to quantify the *rate* at which the system's average recognition score *R* decrease as a function of injury level *p*:

(1) $$R(p)=A-Be^{p}$$

The functional form *R*(*p*) is produced from a linear regression over *N* = 1,080 normalized data points obtained from computational experiments. The values for the constants in Equation (1) are [*A* = 1, *B* = 0.24] for pie-charts from Wang et al. and [*A* = 1.21, *B* = 0.22] for pie-charts from Dikranian et al. See section Materials and Methods and Supplementary Materials for further details.

### 2.2. Deterioration rates for sparsely-encoded uncorrelated memories

**Figure 4:**
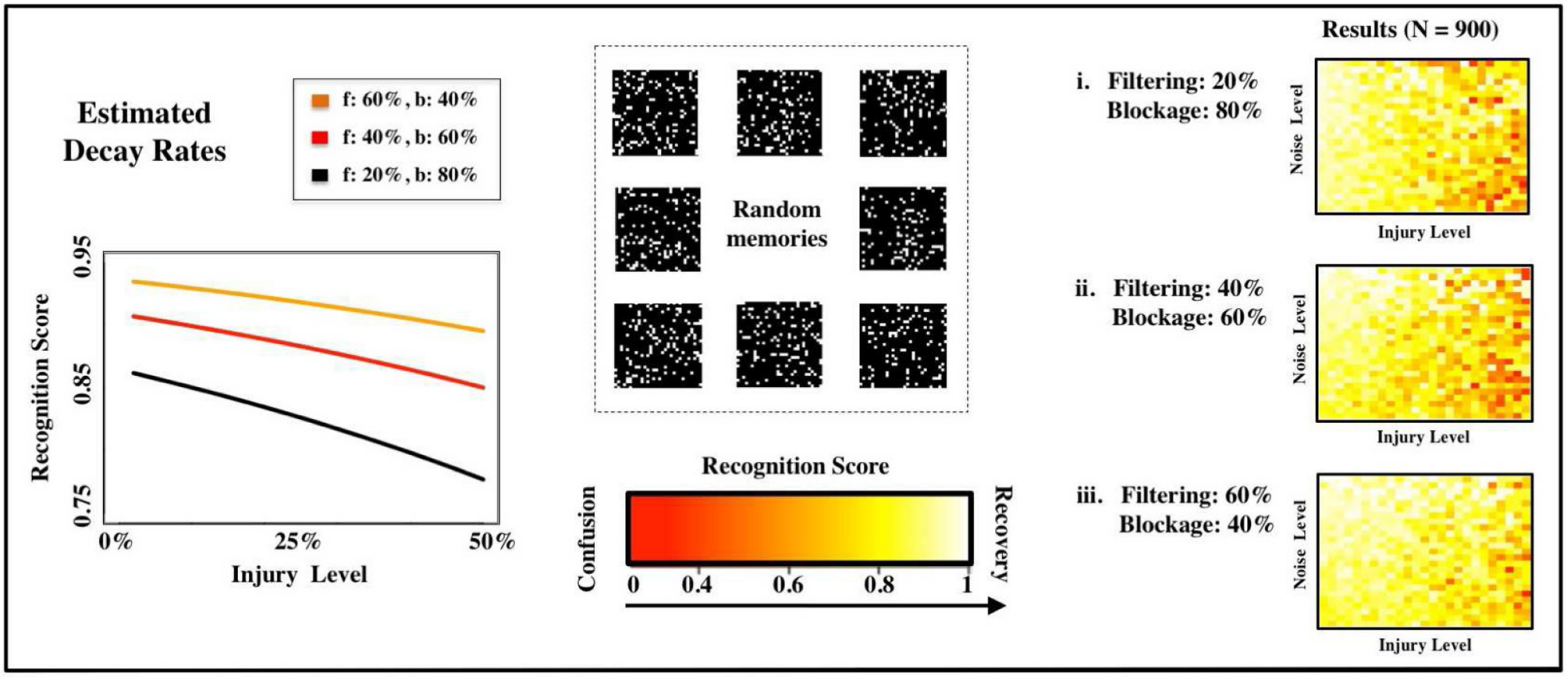
Estimated deterioration rates. We estimate deterioration rates for impairments in memory recognition subject to different distributions of FAS mechanisms (f: filtering in %, b: blockage in %). The computational experiments use random, uncorrelated memories.

After illustrating our model, injury protocols, and performance metrics for a memory-recall task using highly-correlated faces, we move on to a more abstract setup. Consider now a multi-state Hopfield network with *M sparse* and *uncorrelated* memories (see Figure 4), where *M* is now close to the theoretical capacity of the system (*M* ≈ 0.14*N*, see Hopfield, 1984). It is obvious that any plausible *type* of impairment to the individual neurons will ultimately lead to deteriorated recognition scores *R* as a function of *p*. In our experiments, for a fixed noise level, the normalized decay rates take the functional form

(2) $$R(p)=A-Be^{p}.$$

Previous studies of injury studies in Hopfield networks where done in a binary setup, i.e., a neuron was either fully functional or fully impaired. See, for instance, Ruppin and Reggia (1995). In our setup, this would correspond to having a pie-chart with 100% of the FAS in the blockage regime. To contrast past approaches with our own, we vary the contributions of the two most prevalent types of FAS mechanisms (filtering and blockage) in our simulations. The distinct deterioration rates are shown in Figure 4A and summarized in Table 1. The discrepancies are significant and may lead to very different estimates regarding the stage of the disease or its progression. We will discuss this in more depth in the following sections.

**Table 1:** Deterioration rates for sparsely-encoded uncorrelated memories.

**FAS distribution**	**Mean deterioration rates**
Filtering = 60% ; Blockage = 40%	*R*(*p*) = 1−0.06·e^p^
Filtering = 40% ; Blockage = 60%	*R*(*p*) = 1−0.09·e^p^
Filtering = 20% ; Blockage = 80%	*R*(*p*) = 1−0.13·e^p^

## 3. Materials and methods

### 3.1. Modeling dynamical impacts of FAS

Focal Axonal Swellings (FAS) are an ubiquitous pathological feature to several brain disorders (McArthur et al., 2004; Coleman, 2005; Bayly, 2006b; Tang-Schomer et al., 2010, 2012; Xiong et al., 2013). Their presence is known to distort neuronal spike-train dynamics, but precise electrophysiological recordings of pre- and post-FAS spike train dynamics are still unavailable. The recent theoretical models of Maia and Kutz (Maia and Kutz, 2014a,b; Maia et al., 2015), however, provide important estimates of spike train deterioration due to FAS. Using this model, we can characterize the effect of injury on the firing rate activity through a response function. The state variable of a Hopfield node, *S*_*i*_, is equivalent to the firing rate of that node. The collection of all nodes is denoted by the vector **S**. Injuries can then be characterized by the transfer function

(3) $$\tilde{S}=F(S,\beta ).$$

where $\tilde{\text{S}}$ is the effective firing rate (state) after the FAS, **S** is the firing rate (state) before the FAS, and β is a parameter vector indicating one of three injury types applied to individual nodes of the network (Maia and Kutz, 2014a,b). The function *F*(·) maps the pre-injury to post-injury firing rates using biophysically calibrated statistical distributions of injury in both frequency and size (see Supplementary Materials). If no FAS occurs to a given axon, then its state is unaffected (β_0_). This occurs with probability 1 − *p* where *p* is the probability of injury and what we term *injury level*, i.e., larger *p* implies more injury. For those neurons with axonal swellings, the following manifestation of spike train deformations have been observed: unimpaired transmission (β_1_), filtered firing rates (β_2_), spike reflection (β_3_), or blockage (β_4_). The injury type is dependent upon the geometry of the swelling, with blockage being the most severe injury type. From biophysical data collected on injury statistics (Dikranian et al., 2008; Wang et al., 2011), both in swelling size and frequency, we assign a prescribed percentage of each type of injury (β_*j*_) to the network using calibrated simulations of spike propagation dynamics (Maia and Kutz, 2014a,b). For a blockage injury (β_4_), no signal passes the swelling so the effective firing rate of the neuron goes to zero. Thus, $\tilde{\text{S}}=0$ which prevents the neuron from adapting to the collective dynamics. Filtering injuries were taken to decrease the firing rate, with higher firing rates having a stronger chance of decreasing due to pile-up effects in the spike train (Maia and Kutz, 2014b). Reflection of spike trains effectively filters the firing rate of an axon by a factor of two so that $\tilde{\text{S}}=0.5\text{S}$. This is due to the fact that the reflected spike annihilates an oncoming spike (Maia and Kutz, 2014a). Overall then, the method for producing the filtering function *F*(·) uses the most advanced experimental findings to date with recent computational studies of spike train propagation through FAS (Maia and Kutz, 2014a,b). See the Materials and Methods section and the Supplementary Materials for details on how we compute the statistical distribution of injury types that are parametrized by the parameter β_*j*_.

### 3.2. TBI/FAS data from adult rats and infant mice

As in Maia and Kutz (2017), we use TBI/FAS data of adult rats from Wang et al. (2011) to calibrate the distributions of FAS following TBI in our simulations. This allowed us to reconstruct distributions of total area per swelling and their functional deficits (blockage, reflection, and filtering). See Supplementary Materials for details. Additionally, we use Dikranian's morphometric analysis of TBI experiments in infant mice (Dikranian et al., 2008). See Table 2 for the experimental parameters of the two studies.

**Table 2:** Experimental parameters in animal studies by Wang et al. (2011) and Dikranian et al. (2008).

**Study**	**Wang et al**.	**Dikranian et al**.
Animal	adult rats	infant mice
Sample size (per time point)	5	4-6
Injury location	optical nerve	cortex
Time to sacrifice	12/ 24/ 48 h	30 min, 5/ 16/ 24 h

### 3.3. Simulation protocols and parameters

For the simulations, we considered different calibrations of injury mechanisms (see pie charts in Figures S1,S2): Three in the case of Wang et al. and four in the case of Dikranian et al. The distributions were derived by evaluating swelling parameters at different times of sacrifices after the injury occurred (see Supplementary Materials). For each distribution, we performed multiple rounds of simulations, each running over a wide range of injury and noise levels. Recognition scores resulting from the analysis of all simulation results were used to fit decay rates. The analysis was performed for two recognition tasks, using (i) highly-correlated facial images and (ii) sparse, random patterns. The running times of the simulations strongly depend on the type and amount of injury introduced into the network. For larger numbers of neurons and significant levels of injury, the simulations are computationally very expensive. The training itself depends on the number of neurons as *O*(*N*^2^). Further details on the model and its implementation can be found in the Supplemental Material.

The choice of simulation parameters (see Table 3) was guided by balancing between computational expenses and covering a reasonably large parameter range. All code is publicly available on GitHub for possible future studies.

**Table 3:** Experimental parameters used in simulations.

	**Face recognition**	**Random patterns**
*N*	1,080	900
Number of runs	12 per data point	7 per data point
Number of memories	3–5	126
Overlap of memories	~65%	~20−25%
Injury parameters	0–30% (Δ = 0.01)	0–50% (Δ = 0.02)
Noise parameters	0–30% (Δ = 0.01)	0–50% (Δ = 0.02)

## 4. Discussion

Overwhelming experimental evidence suggests that FAS is the hallmark manifestation of injuries on neuronal networks. Moreover, there now exists a wealth of experiments characterizing the statistical distribution of FAS as a function of injury level, including size and frequency of swellings (Coleman, 2005). Statistics can even be collected for specific neurodegenerative diseases such as Alzheimer's (Krstic and Knuesel, 2012), Parkinson (Galvin et al., 1999), or Multiple Sclerosis (Hauser et al., 2006), where the swellings occur as a consequence of complicated biophysical and biochemical deterioration of neurons. These neurodegenerative diseases affect a large portion of adults, with Alzheimer's disease alone estimated to be the third leading cause of death, just behind heart disease and cancer, for older people. Likewise, TBI is responsible for millions of hospitalizations worldwide every year (especially among contact-sport practitioners) and is the leading cause of death among youngsters (Faul et al., 2010; Jorge et al., 2012; Xiong et al., 2013).

In this work, we consider impairments caused by FAS in a Hopfield-like computational model for associate memory. In the face-recognition task, the presence of a significant number of blocked neurons in the network blurs the reconstructed concepts (faces) and decreases the accuracy of the recalled information. Both filtering and reflection regimes lead to confusion of correct states with their neighboring ones (in a Hamming distance sense). This decreases the ability of the network to perform denoising tasks. In most cases, all three impaired regimes (filtering, reflection, and blockage) occur simultaneously in the network (Maia and Kutz, 2014a). An interesting consequence is that injured networks often produce erroneous associations of memories. Specifically, it *confuses* the concepts. Based on simulations of an uninjured network with noise, it could easily be conjectured that the fixed points associated with a given memory would *disappear*, with the system's dynamics simply preventing it from converging to the correct pattern. Instead, we observe that the noise fluctuations cause the dynamics to converge to an erroneous fixed point. For modest amounts of initial noise (15–30%), the affected system confuses the stored patterns and looses the ability to separate them properly. Confusion of concepts is especially pronounced when blockage and filtering account for the majority of FAS effects.

We calibrate our FAS distributions (pie-charts) from two experimental sources (Dikranian et al., 2008; Wang et al., 2011). We believe that the distributions from Dikranian et al. (2008) are more relevant since they damage the cingulum—recognized as fundamental to memory association—instead of the optic nerve. In addition to a TBI protocol better linked physiologically to memory impairments, they provided a distribution plot for the diameters of the spheroids, which allowed us to generate FAS in a much more direct way. See the Supplementary Materials for more details.

Our second associative-memory task was in a more abstract setup, with memories being random and uncorrelated. Previous studies investigated injuries in this kind of network, but in a binary way, i.e., when neurons are either fully injured or fully functional. Binary setups, however, cannot capture the types of frequency-dependent errors demonstrated in current FAS research (Maia and Kutz, 2014b). As a consequence, they cannot handle the nuanced types of deficits observed in TBI and neurodegenerative diseases, and clinicians would likely *underestimate p* from the *R* scores of their patients using the decay coefficient *B* derived from these sources. It is unlikely that estimates obtained from binary injury protocols—where the error produced by a single cell is always maximal—could be applicable to concussions (milder and more common forms of TBI) or early dementia, where successful diagnostics are most needed. In fact, in all seven of our derived FAS pie-charts, injured neurons in blocking regime are <50%, whereas a significant percentage of impaired neurons retain some activity level (reflection, filtering). Distinguishing FAS distributions for different stages of neurodegenerative diseases is especially important in the context of diagnostics, where the focus lies on detecting early signs of a progressing condition.

## 5. Conclusion

Focal Axonal Swellings (FAS) are a hallmark feature of TBI and neurodegenerative diseases such as Alzheimer's, Parkinson's and Multiple Sclerosis. Our study characterizes a neural network's ability to handle noise and perform recognition tasks at different levels of injury. Our model reproduces symptoms commonly observed in patients suffering from brain disorders (Knutson, 2012) arising from the above discussed causes. In the face-recognition task, the injured network loses accuracy in recalling facial images and confuses faces with similar features. From a Hopfield network viewpoint, less accuracy means that fixed points encoding memories loose their stability. At higher injury levels, the network's dynamic is driven away from the correct face encoding, settling closer to a fixed point associated with another facial pattern. Instability of the fixed points and emergence of wrong attractors results from a large percentage of neurons manifesting FAS, leading ultimately to confusion between previously stored images. Performance decline was formalized through estimating *deterioration rates*. for the recognition score. We tested the influence of different distributions of FAS mechanisms on deterioration rates for a set of random, uncorrelated memories. Our results show a significant discrepancy between our proposed multi-mechanism FAS model and compared to the previously studied binary lesion models.

We calibrate FAS parameters in our simulations with experimental TBI data; Dikranian et al. (2008) studied FAS in the cortex of infant mice, whereas Wang et al. (2011) investigated swellings in the optic nerve of adult rats. Although Dikranian et al. (2008) provides detailed morphometric data for injured axons in the infant mice, their functional assessments might be still a distant proxy for memory development in human children (McAllister, 2004). TBI experiments in adult rats (Xiong et al., 2013) report functional impairments more analogous to human patients—like deficits in tasks involving context memory (Schacter, 1987), conditional associative learning (Petrides, 1985), planning (Shallice and Evans, 1978), and other cognitive tasks (McDowell et al, 1997). Adult rats, however, may exhibit memory deficits after mild TBI even without many signs of axonal injury (Lyeth, 1990). In fact, recent studies demonstrate that catecholamines play a central role in the neurochemical activation and regulation of working memory (McAllister, 2004) and such effects were not incorporated in the model. Thus, axonal structural damage may be sufficient but not necessary for the production of neurological and cognitive symptoms associated with TBI. This will be considered in future studies.

There is much room for improvement in our injury model, especially given the variety of effects of different types of brain disorders. The framework introduced in the present article is limited to associate memory. While other forms of memory impairment are also known to occur as a result of neurodegenerative diseases and traumatic brain injury, incorporating these into the model was beyond the scope of this study. Furthermore, we only consider the three basic injurious FAS mechanisms introduced in the Maia and Kutz model and leave out additional physiological effects that might affect the severity of memory impairments. These include the squeezing of neighboring axons when swellings occur in densely connected regions.

Although FAS are universal pathological features, our results should be regarded as a first step towards integrating them into functional neuronal networks and linking cellular impairments to observable psychophysical deficits.

## Author contributions

MW, PM, and JK designed; MW and PM: conducted the study; and MW, PM, and JK: wrote the manuscript.

### Conflict of interest statement

The authors declare that the research was conducted in the absence of any commercial or financial relationships that could be construed as a potential conflict of interest. The reviewer PJT and handling Editor declared their shared affiliation.

## Footnotes

^1^Credit is hereby given to the Massachusetts Institute of Technology and to the Center for Biological and Computational Learning for providing the database of facial images. http://cbcl.mit.edu/software-datasets/heisele/facerecognition-database.html
