# Supplementary material for: Estimating Memory Deterioration Rates Following Neurodegeneration and Traumatic Brain Injuries in a Hopfield Network Model
Source: Front Neurosci. 2017 Nov 9;11:623. doi: 10.3389/fnins.2017.00623 (PMC5684180; doi:10.3389/fnins.2017.00623)
Supplement: Supplementary file 1 [file DataSheet1.pdf]

Supplementary Material: Estimating Memory Deterioration Rates Following Neurodegeneration and Traumatic Brain Injuries in a Hopfield Network Model

Melanie Weber*, **Pedro D. Maia and J. Nathan Kutz**
*Correspondence:
Melanie Weber
melwe@uw.edu, pmaia@uw.edu, kutz@uw.edu

1 HOPFIELD NETWORK MODEL FOR ASSOCIATIVE MEMORY

J. Hopfield made seminal contributions to the study of collective properties that emerge on systems of equivalent components (or neurons). He developed a model to describe content-addressable memory in an appropriate phase space for neuronal networks. The model incorporated aspects of neurobiology and its underlying neuronal circuitry. Within this framework, he was able to study properties such as familiarity recognition, categorization, error correction, and time sequence retention. Our computational model is based on J. Hopfield's original publications (Hopfield, 1982, 1984; Hopfield, Tank, 1985) and more recent extensions (Gerstner et al., 2014; Benna, Fusi, 2015).

1.1 Standard Hopfield Model

In Hopfield's original model, a neuronal network is composed of N neurons that attend binary states $S_i \in \{-1, 1\}$. The connections between neurons are responsible for information transference and processing in the network. They are represented by weights w_{ij} (linking neurons i and j), and stored in a connectivity matrix $W = (w_{ij})$. In this setting, the neuronal states evolve in time according to (Hopfield, 1982, 1984; Hopfield, Tank, 1985)

$$dS_i(t) = \sum_j w_{ij} \cdot g(S_j(t))dt \tag{S1}$$

where the gain function g is given by

$$g(x) = \begin{cases} 1, & x \geq 0 \\ -1, & x < 0 \end{cases} . \tag{S2}$$

The most important property of the model is the ability to encode memories as fixed points of the system. When a noisy input is presented, it converges to the closest fixed point (closest known concept) in a process commonly referred as *memory association*.

1.2 Extended Hopfield Model

Neuronal states are theoretically modeled as continuous spike trains transmitted through axonal channels Adrian (1926); Richmond et al. (1987). In computational studies, these continuous states are discretized

for more efficient computability. Hopfield's original model as described in the previous section considers two binary states distinguishing between an 'on' and an 'off' mode. However, the binary model is not rich enough to model more sophisticated injury mechanism, such as filtering and reflexion in the Maia and Kutz theory. While a continuous model was beyond the scope of this study, we implemented a multi-level discrete state model to account for different modes of neuronal activity. In our extended Hopfield model, neurons may achieve multiple discrete states (Gerstner et al., 2014; Benna, Fusi, 2015)

$$S_i \in \{0, 1, ..., s-1, s\}.$$

The dynamical evolution of the system is also governed by a more sophisticated equation:

$$dS_i(t) = \underbrace{-\tau^{-1} \cdot S_i(t)dt}_{\text{self-dynamics}} + \underbrace{I_i(t)dt}_{\text{external input}} + \sum_j \underbrace{w_{ij} \cdot g(S_j(t))dt}_{\text{input from other neurons}} + \underbrace{\mu \cdot dB_i}_{\text{noise}}\ , \tag{S3}$$

with sigmoid gain function g given by

$$g(x) = 0.5(1 + \tanh(\beta x)). \tag{S4}$$

The constant τ gives the time-scale of the dynamics. Direct inputs for neuron i (e.g. external stimuli) are represented by $I_i(t)$. The term B_i corresponds to a Wiener Process with intensity μ, and is a proxy for stochastic fluctuations in the firing rates. The (continuous) states are ultimately rounded to the nearest discrete state by a scaling function

$$m(t) = \max_{1 \le i \le N} |S_i(t)| \tag{S5}$$

$$\hat{S}_i(t) = \left[\frac{s \cdot S_i(t)}{m(t)} \right] \tag{S6}$$

The resulting stochastic differential equation takes the following form when discretized:

$$S_{i+1} = S_i + \Delta t\ f_1(S_i) + f_2(S_i)\ dB_i \tag{S7}$$

$$\text{with } f_1(S_i) = -\tau^{-1} S_i + \sum_j w_{ij} g(S_j) + I_i\ ,\ \ f_2(S_i) = \mu S_i$$

and Brownian increment $dB_i = B_i(t + \Delta t) - B_i(t)$.

We solved the system numerically using the *Euler-Maruyama Method* (Higham, 2001) and made all our codes available. If higher accuracy is desired beyond the Euler-Maruyama scheme, recent algorithms have been developed to potentially improve accuracy and stability (see (Rößler , 2009) and (Omar et al. , 2011) based on (Milstein , 1975)). For the purposes of this study, such schemes are not required.

For the simulations, we considered the following stopping criterion: The final state is reached, once the states of individual neurons do not change anymore under the network dynamics. Computationally, this is indicated by the absence of significant changes in the overlap function. In our simulations the final state was reached after a small number of time steps due to the relatively small network sizes.

As noted earlier, FAS may alter the activity state of an injured neuron. Thus, a broader class of states is required to study effects of more sophisticated mechanisms. Whereas blockage of spike trains can

be described in binary networks, more complex feature discriminations like reflection and filtering, that transfer partial or altered information, require a multi-state setting. For this, we converted our sample set of facial images (Weyrauch et al., 2004) from originally colored to nine distinct shades of gray ($s = 8$) in addition to the (standard) binary black-white states. With this extension, the computational network can also discriminate images of human faces that naturally share several features (high overlap).

1.3 Earlier Associative Memory Models

A number of early attempts on modeling associative memory with networks predates key ideas in Hopfield's model (McCulloch, Pitts, 1943; Hebb, 1949; Steinbuch, Piske, 1963; Willshaw, 1969; Little, 1974; Kohonen, 1989). Among the very early work is Pitt's 1943 article (McCulloch, Pitts, 1943) that introduces a simple neuron model with binary states $\{0, 1\}$, where output signals (1) indicate an input signal above a certain threshold. Later a similar setup is used by Willshaw et al. (Willshaw, 1969) to describe recurrent neural networks where the output at step t is taken as input in step $t + 1$. A few years before Hopfield's work, Little (Little, 1974) suggested an Ising model – well known from physics – for modeling neural system. In analogy to binary spins, Little's neurons could take the states $\{-1, 1\}$. With his model, Little showed the emergence of persistant states in the neural network.

2 THEORETICAL FRAMEWORK FOR FAS EFFECTS

Maia and Kutz developed in a series of papers a theoretical framework for characterizing the anomalous effects of FAS to spike propagation (Maia, Kutz, 2014a,b; Maia et al., 2015). We review their main results and explain how to add such pathologies (or their proxies) into account for the firing-rate dynamics of neuronal networks (see the schematics in Fig. S1).

The authors distinguish axonal enlargements that lead to minor changes in propagation (β_1) from those that result in critical phenomena such as collisions, reflections or blockage of traveling spikes (β_2, β_3 and β_4). They use three geometrical parameters $(\delta_B, \delta_T, \delta_A)$ to model a prototypical shaft enlargement and characterize all possible propagation regimes in an unmyelinated action potential model. The regimes can be distinguished by evaluating a (simple) function of the FAS geometrical parameters inferred through numerical simulations. They suggest that evaluating this function along axon segments can help detect regions most susceptible to (i) transmission failure due to perturbations, (ii) structural plasticity, (iii) critical swellings caused by brain traumas and/or (iv) neurological disorders associated with the break down of spike train propagation.

Swellings typically delete spikes by a mechanism called *filtering* (β_2), when a first spike changes its profile at the axonal enlargement region and a close second spike interacts with its refractory period. As a consequence, the second spike is deleted in a mechanism of the so-called *pile-up collision* (see (Maia, Kutz, 2014b) for details). Distorted spike trains do not match their corresponding original firing rates (as illustrated in Fig. S1). Instead, they are confused with lower rates, which decrease the system's overall denoising abilities. We simulate the harmful effects of filtering by implementing a statistical version of the confusion matrix from the same source, that in simple terms, evaluates the probability that state i gets confused as state j due to the FAS.

A less frequent mechanism of spike deletion is *reflection* (β_3). There, a traveling pulse is divided into two pulses when it reaches the FAS: one propagating forwards and the other propagating backwards. The backward pulse will collide with the next spike and have them both deleted. Thus, only a fraction of the

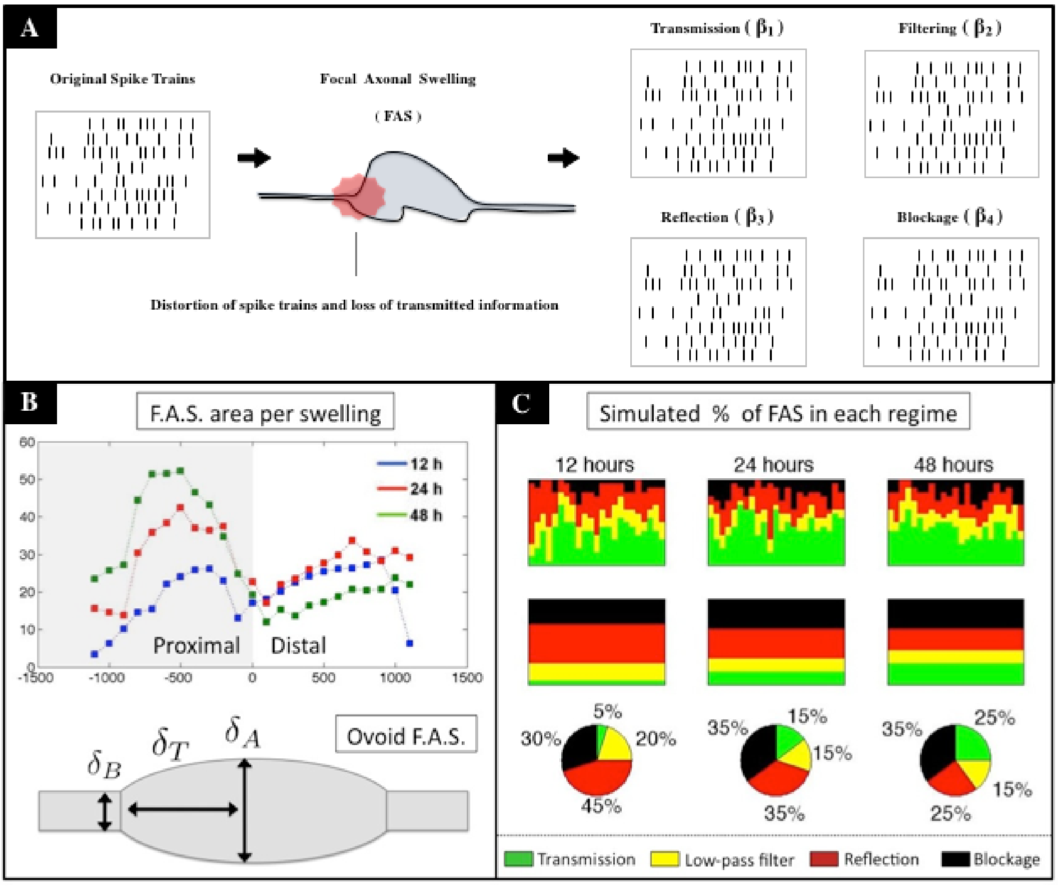

Figure S1. Panel **A:** Schematics for injurious effects of Focal Axonal Swellings (FAS) to spike trains according to the theoretical framework of Maia and Kutz (Maia, Kutz, 2014a,b; Maia et al., 2015). Spike trains may be distorted in four qualitatively distinct regimes depending on geometrical parameters associated to FAS: transmission (β_1), filtering (β_2), reflection (β_3) or blockage (β_4). See references and text for more details. Injuries can then be characterized by the transfer function $\tilde{\mathbf{S}} = F(\mathbf{S}, \beta)$ where $\tilde{\mathbf{S}}$ is the effective firing rate (state) after the FAS, **S** is the firing rate (state) before. Such distortions are incorporated to injured neurons in our Hopfield network simulations in conjunction with experimental morphometric results from Wang et al. (Wang et al., 2011) and Dikranian et al. (Dikranian et al., 2008). Panel **B**: Distribution of functional impairments in injured axons following TBI experiments from Wang et al. (Wang et al., 2011). We generate ovoid/spheroid FAS with areas compatible with the experimental distribution. The geometrical parameters of the FAS define the spike propagation regime. Panel **C**: We generate 12 FAS (column) for each injured axon (row) and order them from worst to best case scenario (upper "flags"). We assume that the worse FAS within an injured axon dominates the others, and classify the entire axon within that category (intermediary "flags"). This leads to the (bottom) pie-charts of impairments for an injured neuronal population. See text for more details.

original encoded information is ultimately transmitted by the spike train. We add this effect in our neuronal network by halving the firing-rate of an injured neuron in this regime.

Figure S1 finally illustrates the *blockage* of spikes (β_4) that occur typically in regions of more dramatic axonal enlargement. In this scenario, no information is transmitted through the damaged axon and the neuron cannot adapt and play its role in the desired collective dynamics: it remains in its initial state. In our neuronal network model, a significant amount of neurons in the blockage regime causes blurs in the reconstructed concepts (memories) and therefore decreases the accuracy of the recalled information. We

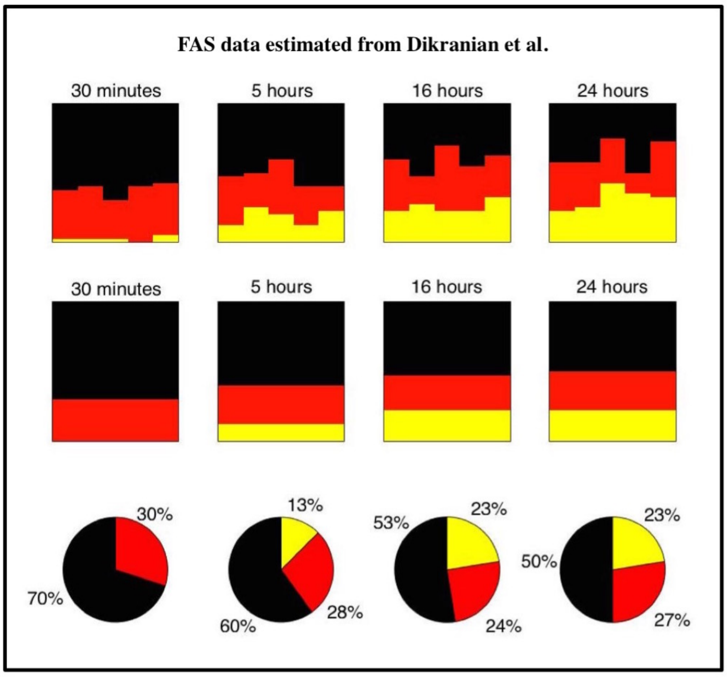

Figure S2. Pie charts illustrating the distribution of functional impairments in injured axons following TBI experiments from Dikranian et al. (Dikranian et al., 2008). We generate ovoid/spheroid FAS following the reported experimental distribution of FAS diameters. The geometrical parameters of the FAS define the spike propagation regime. We generate 5 FAS (column) for each one of the 40 injured axons (row) and order them from worst to best-case scenario (upper “flags”). We assume that the worse FAS within an injured axon dominates the others, and classify the entire axon within that category (intermediary “flags”). This leads to the (bottom) pie-charts of impairments for an injured neuronal population. See text for details.

modeled this mechanism by introducing non-adapting neurons into the network, that keep their (possibly noise-affected) initial state over time.

Traumatic Brain Injuries and neurodegenerative diseases induce FAS with tremendous variety of shapes and, consequently, with different functional deficits regarding spike train propagation. Thus, we consider a *distribution* of different FAS mechanism where fractions of neurons are affected by blockage, reflection and filtering (confusion) respectively.

3 IMPLEMENTATION OF MEMORY STORAGE

To simulate a face recognition task, the set of memories has to be *learned* by the network. For this, we encode them in the weights of the neuronal connections as specified by the *weight matrix* of the network:

We consider a system of weighted neurons. The strength w_{ij} of the connection between neuron *i* and neuron *j*, described by the weight of the respective edge, characterizes the information transfer from i to j. Stored in the connectivity matrix $W = (w_{ij})_{1 \leq i,j \leq N}$, they characterize the network's dynamics and encode the set of known concepts corresponding to the system's fixed points.

The weight matrix is constructed from the training set of memories represented as network states:

$$C := \begin{Bmatrix} \vdots & \vdots & \dots & \vdots \\ \text{face 1} & \text{face 2} & \dots & \text{face M} \\ \vdots & \vdots & \dots & \vdots \end{Bmatrix} \rightarrow W = C^T C \tag{S8}$$

The theoretical storage capacity of a (Standard) Hopfield network of size N is $0.14N$ random patterns. In this study, we use a much smaller set of memories, respectively five and three. This is due to the fact, that we store highly correlated facial images as opposed to random patterns. They have a pairwise overlap of 60% due to the structural similarity of faces. The high correlation of the memories significantly decreases the storage capacity and therefore requires the choice of a small set of memories. We choose a setting with highly correlated memories to demonstrate the effects of memory confusion arising from FAS as described earlier.

4 GENERATING RANDOM, UNCORRELATED MEMORIES

We want to generate a set of M random memories to initialize a Hopfield network with N neurons with a size of about the theoretical capacity of the network ($M \approx 0.14N$ Hopfield (1984)). This can be achieved with the following algorithm:

1. Generate a random $N \times M$ pattern matrix P.
2. Apply a predefined threshold to achieve a desired level of sparsity ($P_{\text{thresholded}}$) and update the pattern matrix
$$\hat{P} = P \cdot P_{\text{thresholded}} \; .$$
3. Construct weight matrix as
$$W = P^T \cdot P \; .$$

In our computational experiments, we set $N = 900$, $M = 126$ and $threshold = 1.5$, which yields a sparsity of about 13% in matrix P and a conditionl number of 49.28 .

5 RECOGNITION SCORE FOR NETWORK PERFORMANCE

We developed a recognition score that measures recognition abilities with respect to significance and accuracy in recalling previously stored memory patterns (see Fig. S3).

We assume the existence of an *ideal observer* (cf. Benna and Fusi(Benna, Fusi, 2015)), that knows the whole set of memories and the original pattern underlying the current noisy input. Our recognition score takes the place of this observer by evaluating the current network state against all memorized patterns. In what follows, we describe the computational steps of the recognition algorithm:

(i) *Overlap:* We determine the overlap between the current network state $\{\hat{j}\}$ and the set of stored memories $\mu = 1, ..., M$ by calculating the respective overlap $m^\mu \in \{0, 1\}$ of individual neuronal states $\{j\}$:
$$m^\mu = \frac{1}{N} \sum_j \delta_{|j-\hat{j}|<1}$$

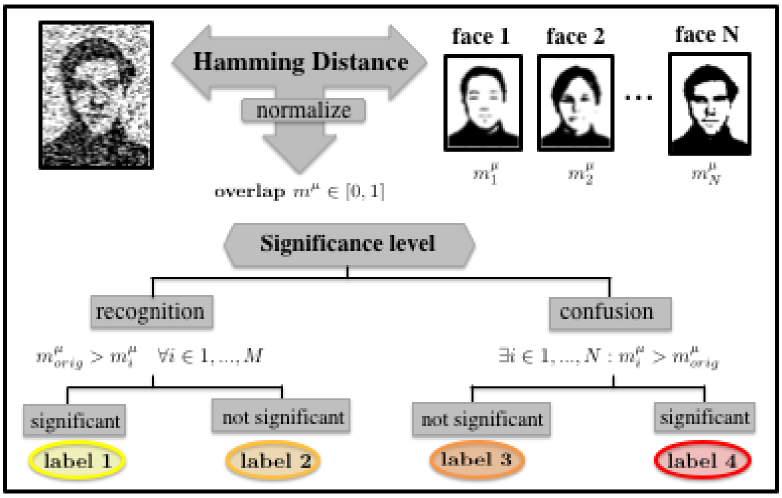

Figure S3. Calculation of recognition scores for measuring memory performance (see *Hopfield Recognition Toolbox*, current version available at GitHub: `https://github.com/MelWe/hopf-recognition`). We use the Hamming distance m_i^μ to measure the overlap between the current network state and the fixed points corresponding to known facial images. Confusion or recognition is characterized by m_i^μ: if the overlap with the correct facial image is highest, we speak of *recognition*, otherwise of *confusion*. A threshold for the difference between the highest and second highest overlap determines whether the recognition or confusion was significant. According to this classification, we assign color labels to each trial which can be displayed in a heat map.

(ii) *Recognition and Significance:* After a pre-defined number of time steps (system's parameter), the network's states are matched to the closest pattern, i.e., we determine the $\mu \in 1, ..., M$, such that

$$d_\mu = |m^{orig} - m^\mu| \text{ is minimal.}$$

If the output pattern matches the original one ($\mu \equiv orig$), we say that *recognition* occurs. Otherwise, we speak of *confusion* of the memories (concepts). The classification is considered *significant* only if

$$|d_\mu - d_i| < t \quad \forall i = 1, ..., M; \ \ (\mu \neq i),$$

where t is a threshold parameter. With this scheme, we classify the memory recall into four groups and assign (numerical) labels.

(iii) *Evaluation:* The recognition score was developed to evaluate the memory performance of our Hopfield neuronal network model over a broad range of injury (parameter *inj*) and initial noise (parameter *noise*). For each pair of parameters $(inj, noise)$ we calculate the score as value of the significance label scaled by the accuracy of the recognition (overlap m^μ).

The final result is a heat map (see Fig. 2,3 in main text) that links recognition score, memory performance and noise handling to different levels of injury.

Code available on **GitHub**: MelWe/hopf-recognition

REFERENCES

Gerstner W, Kistler WM, Naud R, Paninski L (2014) Neuronal Dynamics: From Single Neurons to Networks and Models of Cognition. *Memory and attractor dynamics*, (Cambridge University Press) p.442–66.

Higham DJ (2001) An Algorithmic Introduction to Numerical Simulation of Stochastic Differential Equations, *SIAM Review* 43(3), p. 525-546.

McEliece RJ, Posner EC, Rodemich ER, Venkatesh SS (1987) The Capacity of Hopfield Associative Memory, *IEEE Trans. on Inf. Theory* vol. IT-33(4), p. 461–482.

McCulloch WS, Pitts W (1943) A logical calculus of the ideas immanent in nervous activity, *Bull. Math. Biophys.*, vol. 5, pp. 115–133.

Hebb DO (1949) The Organization Of Behaviour, *Wiley*.

Benna MK, Fusi S (2015) Computational principles of biological memory. *arXiv:1507.07580v1*

Hopfield JJ (1982) Neural networks and physical systems with emergent collective computational properties, *Proc. Nat. Acad. Sci.* (USA) 79, p. 2554–2558.

Hopfield JJ (1984) Neurons with graded response have collective computational properties like those of two-sate neurons, *Proc. Nat. Acad. Sci.* (USA) 81, p. 3088–3092.

Hopfield JJ, Tank DW (1985) Neural computation of decisions in optimization problems, *Biological Cybernetics* 55, p. 141–146.

Kohonen T (1989) Self-organization and Associative Memory: 3rd Edition. *Springer-Verlag New York, Inc.*, New York, NY, USA.

Steinbuch K, Piske UAW (1963) Learning Matrices and Their Applications. *IEEE Transactions on Electronic Computers*, vol. EC-12(6), pp. 846–862.

Little WA (1974) The existence of persistent states in the brain. *Math. Biosc.* no. 19, pp. 101-120.

Willshaw DJ, Bunemann OP, Longuet-Higgins HC (1969) Non-holographic associative memory. *Nature* no. 222, pp. 960-962.

Dikranian K, Cohen R, Mac Donald C, Pan Y, Brakefield D, Bayly P, Parsadanian A (2008) Mild traumatic brain injury to the infant mouse causes robust white matter axonal degeneration which precedes apoptotic death of cortical and thalamic neurons. *Exp. Neuro.*, vol. 211, pp. 551–560.

Wang J, Hamm RJ, Povlishock JT (2011) Traumatic axonal injury in the optic nerve: evidence for axonal swelling, disconnection, dieback and reorganization, *Journal of Neurotrauma* 28(7), pp. 1185–1198.

Weyrauch B, Huang J, Heisele B, Blanz V (2004) Component-based Face Recognition with 3D Morphable Models, *First IEEE Workshop on Face Processing in Video (Washington, D.C.).*

Maia PD, Kutz JN (2014) Compromised axonal functionality after neurodegeneration, concussion and/or traumatic brain injury, *J Comput Neurosci* 37, pp. 317-332.

Maia PD, Kutz JN (2014) Identifying critical regions for spike propagation in axon segments, *Journal of Computational Neuroscience*, vol. 36(2), pp. 141-155

Maia PD, Hemphill MA, Zehnder B, Zhang C, Parker KK, Kutz JN (2015) Diagnostic tools for evaluating the impact of Focal Axonal Swellings arising in neurodegenerative diseases and/or traumatic brain injury. *J Neurosci Methods*, vol. 253, pp. 233–43.

Adrian ED (1926) The impulses produced by sensory nerve endings. *The Journal of physiology.* vol. 61(1), pp. 49–72.

Richmond BJ, Optican LM, Podell M, Spitzer H (1987) Temporal encoding of two-dimensional patterns by single units in primate inferior temporal cortex. I. Response characteristics. *Journal of Neurophysiology*, vol. 57 (1), pp. 132–146.

Rößler A (2009) Second Order RungeKutta Methods for It Stochastic Differential Equations. *SIAM Journal on Numerical Analysis*, vol. 47(3), pp. 1713-1738.

Milstein GN (1975) Approximate Integration of Stochastic Differential Equations. *Theory of Probability and Its Applications*, vol. 19(3), pp. 557–562.

Omar MA, Aboul-Hassan A, Rabia SI (2011) The composite Milstein methods for the numerical solution of Ito stochastic differential equations. *Journal of Computational and Applied Mathematics*, vol. 235(8), pp. 2277–2299.
